# Comprehensive impedance investigation of low-cost anion exchange membrane electrolysis for large-scale hydrogen production

**DOI:** 10.1038/s41598-020-80683-6

**Published:** 2021-01-11

**Authors:** Immanuel Vincent, Eun-Chong Lee, Hyung-Man Kim

**Affiliations:** Power System and Sustainable Energy Laboratory, Department of Nanoscience and Engineering, INJE University, 607 Eobang-Dong, Gimhae-si, Gyongsangnam-do 621-749 Republic of Korea

**Keywords:** Hydrogen energy, Fuel cells, Renewable energy, Electrochemistry

## Abstract

Anion exchange membrane (AEM) electrolysis is a promising solution for large-scale hydrogen production from renewable energy resources. However, the performance of AEM electrolysis is still lower than what can be achieved with conventional technologies. The performance of AEM electrolysis is limited by integral components of the membrane electrode assembly and the reaction kinetics, which can be measured by ohmic and charge transfer resistances. We here investigate and then quantify the contributions of the ohmic and charge transfer resistances, and the rate-determining steps, involved in AEM electrolysis by using electrochemical impedance spectroscopy analysis. The factors that have an effect on the performance, such as voltage, flow rate, temperature and concentration, were studied at 1.5 and 1.9 V. Increased voltage, flow rate, temperature and concentration of the electrolyte strongly enhanced the anodic activity. We observed that here the anodic reaction offered a greater contribution to the overpotential than the cathode did.

## Introduction

Electricity production by renewable sources such as solar, wind and tidal hydraulics now offers the most promising solutions to our current energy demands, taking a clean environment into consideration^1^. However, the electricity produced directly from renewable sources, such as wind and solar, may be negatively impacted by fluctuations in relevant geographical factors, such as cloud cover and low winds^2^. This then leads to an interrupted supply of the renewable energy, hence renewable energy must be stored and then used on demand for specific applications^2^. Among the various energy storage technologies, storage in the form of hydrogen is considered most preferable, due to the ability to store large amounts of energy for short and long periods of time, which can be decoupled upon demand^3^.

Low-temperature water electrolysis is one of the cutting edge technologies for the sustainable conversion of hydrogen from renewable energy, using water. This technology offers adequate energy storage and grid-balancing utility in power-to-gas operations^4^. The advantages offered by low-temperature water electrolysis include its high efficiency, high product purity, stable output, the feasibility of large-scale production and the capability of incorporating renewable energy as power source^5^.

Currently, the main commercially available water electrolysis technologies are proton exchange membrane (PEM) electrolysis and alkaline electrolysis. A PEM electrolysis performance of 3000 mA cm^–2^ at 1.8 V has been reported (2015)^6^. However, the acidic environment required in PEM electrolysis limits the choice of catalysts to the expensive noble metals, such as platinum, iridium and it oxides^7^. Furthermore, the Nafion-based PEM and titanium stack components directly increase the capital cost of the electrolysis process, hence hindering the wider application of this technology.

On the other hand, we do have alkaline electrolysis that is a mature and less expensive technology, but it cannot be linked with the renewable energies (solar, wind, etc.) for power generation owing to its inability to maintain high-pressure hydrogen, because of the required use of a porous diaphragm and liquid electrolyte^8^.

### Anion exchange membrane electrolysis

**Figure 1:**
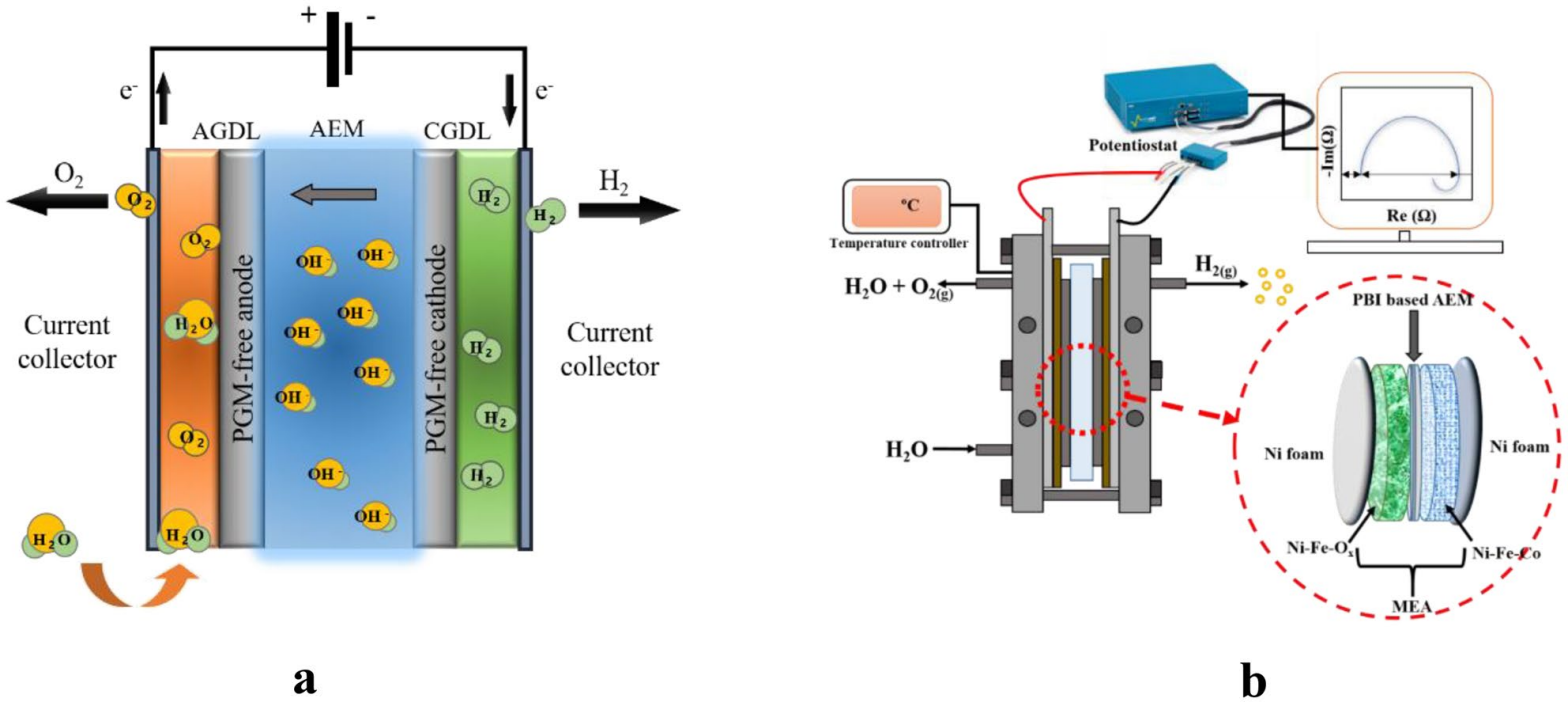
**(a)** Schematic of anion exchange membrane (AEM) electrolysis. *AEM* anion exchange membrane, *AGDL* anode gas diffusion layer, *CGDL* cathode gas diffusion layer. (**b)** Schematic diagram of AEM water electrolysis with EIS experimental setup.

Recently, researchers have developed an emerging third-generation technology, anion exchange membrane (AEM) water electrolysis, which integrates the benefits of both conventional PEM and alkaline electrolysis^9–11^. The AEM electrolysis technology adopts low-cost catalytic materials, as in alkaline electrolysis, and a solid polymer electrolyte architecture, as in PEM electrolysis technology^12^. A schematic of AEM electrolysis is shown in Fig. 1a. AEM electrolysis technology operates in an alkaline environment (pH ~ 10), making it possible the use modest non-noble-metal electrocatalysts, whilst accommodating a zero-gap architecture^13^. The membrane used in this type of electrolysis is a polymeric membrane, containing quaternary ammonium salts. It is relatively inexpensive and has low interaction with atmospheric CO_2_^14,15^. Thus, it is expected that this electrolysis technology should offer better performances and at a lower overall cost^16^.

We have previously reported on performances achieved with AEM electrolysis^17^. In an earlier study of ours, we demonstrated a new membrane electrode assembly (MEA) combination; it comprised a polybenzimidazole (PBI) AEM, and Ni–Fe–O_x_ (for the OER, the oxygen evolution reaction) and Ni–Fe–Co (for the HER, the hydrogen evolution reaction). The best performance was obtained at 1000 mA cm^−2^, 1.9 V and 60 °C, an AEM electrolysis performance of 74% was recorded^17^. Although such AEM electrolysis performance is considered acceptable, it is still lower than that achieved with conventional PEM electrolysis^12,13^. To further advance in this field, it is mandatory to gather more information on the factors that may limit the performance of AEM electrolysis^18^. Accordingly, further consideration/evaluation of various factors are required, e.g., the OER and HER reaction mechanisms, and resistances offered by each of the integral parts/components of the MEA^19,20^. In addition, the following should also be considered: the rate-determining steps of AEM electrolysis, the feasible circumstances of mass transport limitations and degradation of catalytic layer and AEM involved in the electrochemical reaction.

The performance of an AEM electrolyser is directly measured by means of polarization curves (I–V)^21,22^. Such curves offer a reflection of the macroscopic behavior of the whole AEM electrolyser, but reveal no precise information about the effect of the inner components and kinetics of the electrolysis reaction.

In AEM electrolysers, the current expresses the rate of hydrogen production and the voltage is the driving force for the electrolysis reaction. When a voltage is applied between the anode and cathode, the electrons flow through an external circuit, balanced by OH^–^ ion transfer through the AEM, thus the electrolysis takes place^23^. Upon the application of higher voltage, the electrons try to flow through the external circuit and the OH^–^ ions attempt to cross the AEM. However, due to internal barriers, the electrons and OH^–^ ions are not able to transfer at lower voltages^24^. Hence, there is a decrease in hydrogen production due to the voltage drop—this is referred to as the activation overpotential or charge transfer resistance (R_CT_). However, if the applied voltage is relatively low, the flow of electrons is limited by the internal components, such as the AEM, the catalyst layer and the gas diffusion electrode (GDL)—this is referred to as the ohmic resistance (R_ohm_)^25^.

Thus, the performance of an AEM electrolyser depends on three factors: ohmic resistance, kinetic resistance and mass transfer resistance^26,27^. These are sources of overall polarization losses, and they can be separated, and quantified, by the electrochemical impedance spectroscopy (EIS) characterization technique. The EIS technique has therefore been widely applied in various electrochemical processes, such as in batteries and fuel cells, and in PEM electrolysis^28,29^.

AEM electrolysis technology, being a recent and still developing technology, has been little investigated using EIS. EIS investigations could be very useful in terms of contributing to a better understanding of the impedances and kinetics of AEM electrolysis. In this study, we utilized EIS in our broad investigations into AEM electrolysis, in efforts to achieve a better understanding of the resistances involved in AEM electrolysis technology.

The goal of our study was therefore to investigate the various resistances involved in AEM electrolysis, both qualitative and quantitatively. More specifically, we wished to determine the individual resistance contributions towards the overall resistance and the rate-determining step of AEM electrolysis. We looked into determining the effect/s of factors that affect the performance of AEM electrolysis, such as the flow rate of the liquid electrolyte, the electrolyte concentration and temperature under various operating conditions. Finally, we carried out stability tests in an effort to determine whether the performance degradation could be evaluated by EIS at constant current.

On completion of our study, we concluded that the contributions of individual resistances, such as anodic, cathodic, ohmic and charge transfer resistances, are all factors that affect AEM electrolysis performance. Furthermore, we demonstrated, for the first time, how the liquid electrolyte flow rate also affects the performance.

### Theory

#### Nyquist plots

**Figure 2:**
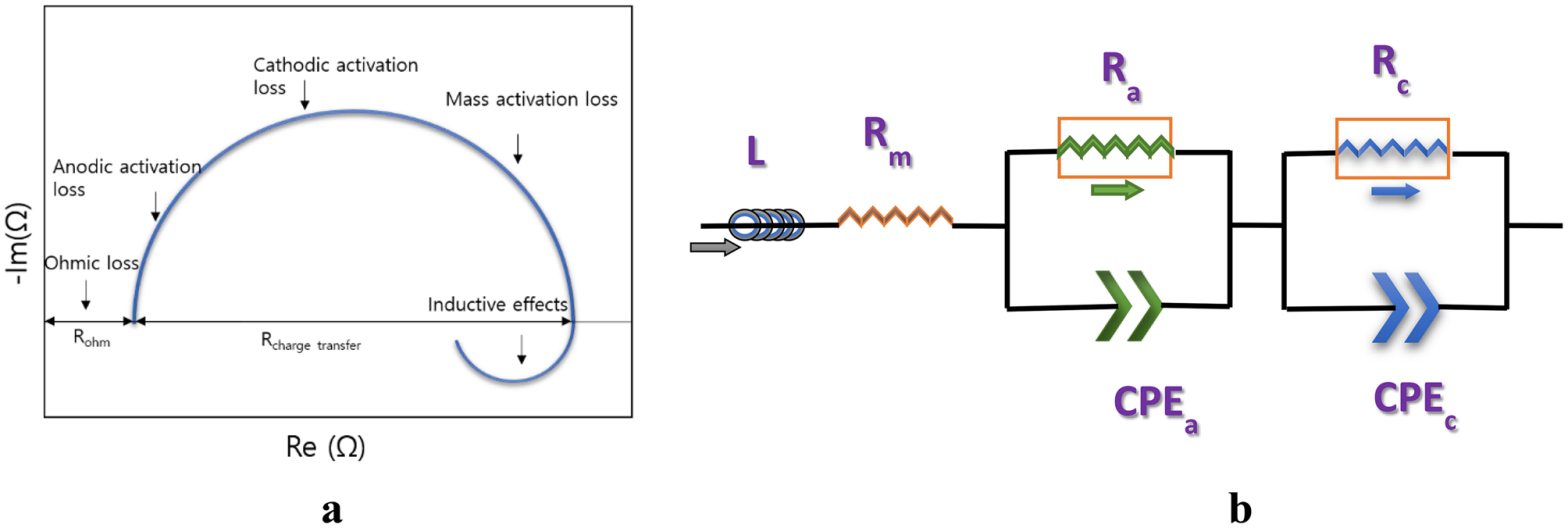
** (a)** Typical Nyquist plot observed for an electrolyser. (**b**) Equivalent circuit model for a single AEM electrolyser. *Rm* membrane resistance, *Ra* anode resistance, *Rc* cathode resistance, *CPEa* and *CPEc* constant phase elements of anode and cathode.

The outcomes of EIS data can be conferred in two ways: a Nyquist plot, or Cole–Cole plot and Bode plot. The Nyquist plot (a parametric plot of the frequency response) is commonly used to determine the stability of a system; it is usually illustrated as a semicircle. It exhibits straight lines at low frequency and a semicircle loop at high frequency. The structure of the curve and arc provides insight into the behavior of electrochemical reactions or dominant phenomena^30^. Figure 2a shows a typical Nyquist plot, wherein the impedances are expressed in the real part (x-axis) and the imaginary part (y-axis) in the semicircle.

The impedance at high frequency, intercepting the x-axis, is the ohmic resistance. The difference between the higher frequency intercept and the lower frequency intercept is the polarization resistance, or the diameter of the semicircle. The charge transfer resistance is overwhelmed by the kinetics of the reaction^31^. According to literature^32^, the concentration polarization resistance is the second arc at the lower frequency. The total resistance of the cell is the summation of resistances at lower frequency and the differential resistance character of polarization curves^33^.

### Equivalent circuit modelling

EIS only measures the responses of electrochemical devices, it does not provide direct phenomenological measurements^34^. An equivalent circuit model has therefore been developed to interpret EIS responses in terms of phenomenological measurements^35^. Equivalent circuit modelling is used to elicit a physical understanding of an electrochemical cell.

The equivalent circuit comprises various components, such as an inductor, a resistor and constant phase elements. In the equivalent circuit model, the resistors constitute the major part; they indicate the ion conduction pathway of ions and electron transfer^36^. This model shows the magnitude of the resistance of a material, for the interfacial charge transport, such as the resistance of a conductor to electron transport or an electrolyte to ion transport. Furthermore, it indicates the resistance to the charge transfer process at the surface of an electrode. The capacitors and inductors represent the space-charge polarization regions, such as adsorption/desorption processes and the electrochemical double layer at electrode interfaces^36,37^. Inductance usually refers to the cables and connectors that are used when recording measurements. The components of the equivalent circuit, such as resistor, inductor and capacitor, are arranged in series and parallel. The constant phase element represents the roughness of the electrode surface, and indicates the OER anodic charge transfer and HER cathodic charge transfer processes, including the mass transfer processes^37^. The equivalent circuit used in this study is shown in Fig. 2b.

## Discussion

### Selection of operating regime of an AEM electrolyser for investigation by EIS

The operation of electrolysis at higher current density means higher hydrogen production^38^, which is beneficial in terms of the capital cost of electrolysis. On the other hand, the operation of electrolysis at higher current reduces the voltage efficiency and increases component degradation. Such degradation and a lower voltage efficiency directly increase the cost of hydrogen production from water electrolysis, the overall production cost of hydrogen is increased^39^.

In this context, for any practical application an electrolyser should operate at the optimal current density (balancing the capital cost and operating cost). However, in our study of the application of EIS to low-cost anion exchange membrane electrolysis, we were particularly interested in operating at lower current density because our future goal was to eventually see the commercialization of an AEM electrolyser that has low cost and a lower catalyst loading of catalyst, without the associated negative effects on the stability and performance. On the other hand, electrochemical impedance spectra at higher current density provide information on the limiting step at nominal current density. (Experimental details pertaining to the AEM electrolysis are described in the experimental section).

### Effect of applied voltage on the resistances

#### Effect of lower voltages on resistances

**Figure 3:**
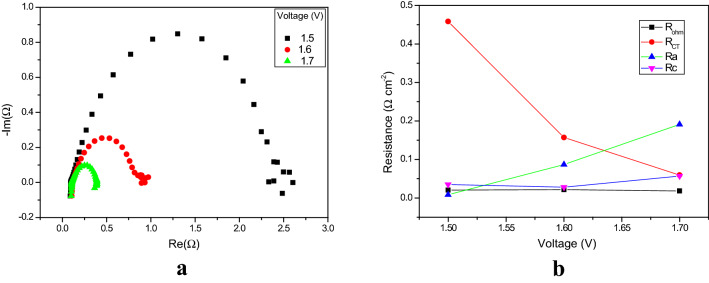
** (a)** Nyquist plots of the EIS measurements (from 100 kHz to 1 MHz), at amplitude 50 mA, at lower voltages. (**b)** Variation in the electrochemical resistances as a function of lower voltages.

The effect of lower voltages on the resistances is very important in effort to understand the catalyst activity. To better understand the activity of the catalyst layer itself on the GDL, experiments were carried out at close to the thermoneutral voltage of electrolysis (1.48 V). The lower voltage EIS experiments were carried out at the following voltages: 1.5, 1.6 and 1.7 V. Figure 3a shows the Nyquist plots of EIS measurements at these low voltages. The high frequency is plotted towards the left side of the plane and the lower frequency towards the right side. Here, a perfect semicircle was obtained, the height and the diameter of which decreased with increasing applied voltage. The decrease in diameter indicates a decrease in the charge transfer resistance. The migration of the ions and electrons is responsible for the transfer of charge at the electrode–membrane interface. Hence, when the applied voltage is increased from 1.5 to 1.7, more ions and electrons pass through the electrode and membrane, hence increasing the rate of reaction, which is directly proportional to the applied voltage. At the lower frequency, there is a small inverted semicircle after the large semicircle. The observation of an inverted semicircle, or low-frequency inductive loops, is attributed to nonstationary behavior. The inductive loops that are observed are due to changes in certain (experimental) factors, such as liquid electrolyte concentration, membrane thickness, hydrogen pressure, and the OER/HER kinetics. There are two possible reasons for the observation of low-frequency inductive loops in the impedance responses:^40^ (1) relaxation of adsorbed intermediates species associated with cathodic reactions and the formation of hydrogen peroxide, and (2) formation of NiO with subsequent dissolution of Ni from cathode catalyst layer. The charge transfer resistances for the 1.5, 1.6 and 1.7 V experiments are 458.6, 157.2 and 59.6 mΩ cm^–2^. The corresponding three ohmic resistances (R_ohm_) are 20.6, 21.6 and 18.2 mΩ cm^–2^. As is evident in Fig. 3b, there is not much change in the R_ohm_.

The rate of reaction at lower cell voltage is low (the rates of oxidation and reduction are both low); much energy is required to overcome the activation barriers, to move electrons through the external circuit and achieve OH^–^ ion transport through the membrane^41^. Due to the higher energy requirement, the charge transfer resistance is drastically reduced when the voltage was changed from 1.5 to 1.6 V; it was reduced by 65%. Thereafter, the when the voltage was changed from 1.6 to 1.7 V, the reduction was 62%. The ohmic resistance was not much influenced by the lower voltages. The anodic and cathodic resistance began to increase from 1.5 to 1.7 V, which indicated the commencement of the redox reaction. However, it is expected that when the voltage is increased, the charge transfer resistances will begin to decrease (as observed when the voltage was increased from 1.5 to 1.6 V). Evidence hereof is also seen in the decrease in diameters of the semicircles of the Nyquist plots. Charge transfer resistance decreased with increasing cathodic voltage at low voltages but it remains constant at high voltages. This result indicates that the electrochemical reduction reaction is controlled by the charge transfer process at lower voltage.

#### Effect of higher voltages on resistances

**Figure 4:**
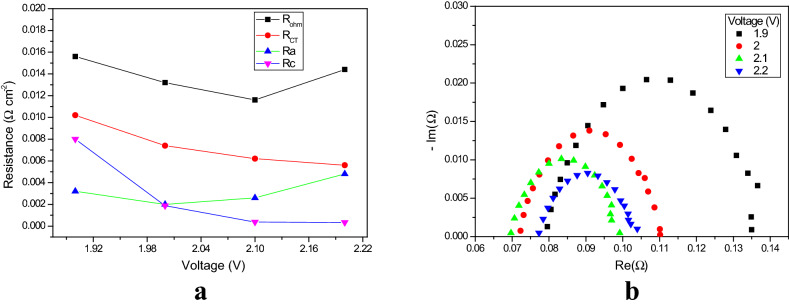
** (a)** Nyquist plots of EIS measurements (from 100 kHz to 1 MHz), at amplitude 50 mA, as a function of higher voltages. (**b)** Variation in the relevant electrochemical resistances as a function of higher voltages.

The higher voltage region is the operating voltage region of AEM electrolysis; it is in the range 1.8–2.2 V. Electrolysis experiments were carried out at higher voltages, specifically, 1.9, 2, 2.1 and 2.2 V. The Nyquist plots of the EIS measurements from the higher voltages are shown in Fig. 4a. All these plots are well-shaped semicircles; the semicircle moves to the left (higher frequency) when the voltage is increased. The diameter of the semicircle decreases when the voltage is increased.

The ohmic resistances for the 1.9, 2, 2.1 and 2.2 V experiments are 15.6, 13.2, 11.6 and 14.2 mΩ cm^–2^, respectively, and the charge transfer resistances 10.2, 7.4, 6.2 and 5.6 mΩ cm^–2^ (see Fig. 4b), respectively. However, the ohmic resistance dominates over charge transfer resistance. The ohmic resistance from 1.9 to 2.1 V decreased from 15.6 to 11.6 2 mΩ cm^–2^; beyond 2.1 V it increased, to 14.2 mΩ cm^–2^ at 2.2 V. The reduction in ohmic resistance from 1.9 to 2.1 V suggests some restructuring of the polymer during operation, or membrane thinning, since this region is the membrane resistance dominant region^42^. The increase of ohmic resistance beyond 2.1 V may be associated with bubble formation or deterioration of the catalytic layer^43^. The charge transfer resistance decreased with increasing voltage. At higher voltage, the rate of the redox reaction is very high, which then decreases the charge transfer resistances with increasing voltage. The increase in the anodic resistance is the major contributor to the increase in the overall ohmic resistance (see Fig. 4a). Hence, increasing the operating voltage beyond 2.1 V may adversely affect the components of the MEA in an AEM electrolyser.

### Effect of flow rate on resistances

In AEM electrolysis, a KOH electrolyte solution, with water molecules carrying OH^–^ ions, is circulated to the anode side of the electrolyser. The liquid electrolyte hydrates the membrane and the catalyst layer on the GDL without an additional supply of (excess) water. The flow of liquid electrolyte is very important as it is related to the mass transfer of the redox reaction. The flow rate has a direct effect on the AEM electrolysis performance, hence it is important to understand the effect of flow rate on the various resistances.

**Figure 5:**
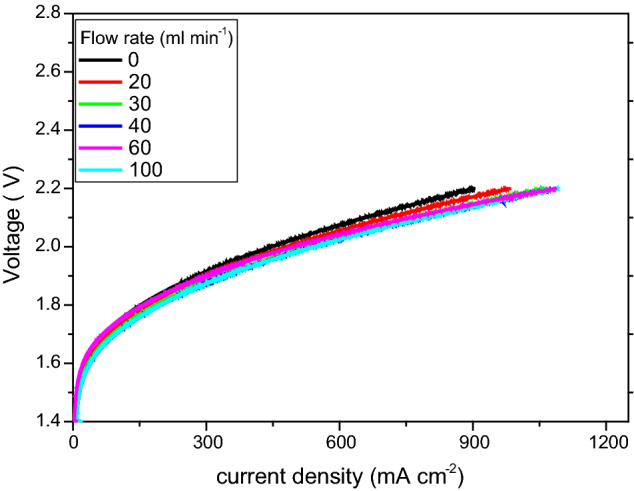
Polarization curves of an AEM electrolyser, recorded for different flowrate of KOH electrolyte, at 60 °C.

In our investigations, the liquid electrolyte (1 M KOH) flow rate was varied from 50 to 110 mL min^–1^, applying increments of 20 and 40 mL min^–1^. Electrochemical impedance spectra were recorded for the respective flow rates. Figure 5 shows the performances recorded for the different flow rates. The resistances were derived after application of the equivalent circuit model.

**Figure 6:**
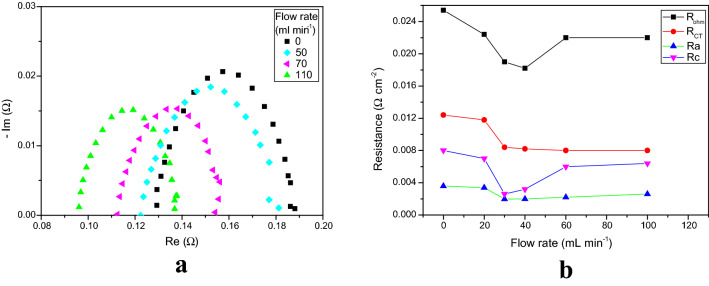
** (a)** Nyquist plots of EIS measurements (from 100 kHz to 1 MHz) from the function of liquid electrolyte flow rate at 1.9 V. (**b)** Variation in the relevant electrochemical resistances as a function of flow rate at 1.9 V.

Figure 6a shows Nyquist plots of EIS measurements, revealing the effect of the electrolyte flow rate (at 1.9 V). Figure 6b shows variations in relevant electrochemical resistances as a function of flow rate (at 1.9 V). The perimeter height and diameter of the semicircle increased with decreasing flow rate. When the flow rate was increased, the diameter and perimeter height decreased. Figure 6b shows that the ohmic resistance is the dominating contact resistance (at all flow rates here). The difference between the ohmic resistance and the charge transfer resistance is very high. When the flow rate was increased from 0 to 70 mL min^–1^, both the ohmic resistance and charge transfer resistance decreased. The ohmic resistance decreased from 25.4 to 18.2 mΩ cm^–2^ (28.2% reduction). However, after 40 mL min^–1^, there was no significant change in the contact resistance, but the ohmic resistance increased from 18.2 to 22.2 mΩ cm^–2^ (20% increase).

The following reason is offered for the decrease in ohmic and charge transfer resistances (flow rate < 40 mL min^–1^). During electrolysis operation, in the redox reaction, bubbles of oxygen form on the anode and hydrogen on the cathode. These products must be removed immediately from the surface of the GDL to prevent them blocking the catalyst active sites. This can be achieved by increasing the flow rate of the electrolyte. Internal forces help to keep the two gases apart^42^. On the other hand, a higher flow rate rapidly removes the OH^–^ ions, hence reducing the available reaction time for oxidation and reduction, subsequently leading to a lower availability of OH^–^ ions to the catalyst, and then a significant increase in the overpotentials (especially for the OER). Figure 6b shows that, when the flow rate increased, the anodic resistance increased from 18 to 22 mΩ cm^–2^. The overall ohmic resistance increased at flow rates > 40 mL min^–1^.

### Effect of KOH concentration on performance and resistances

**Figure 7:**
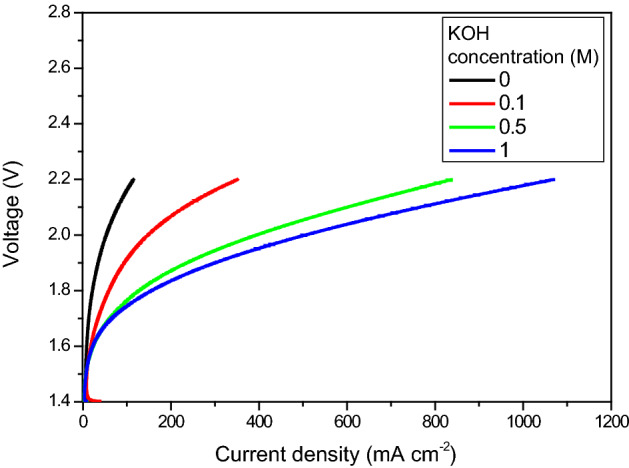
Polarization curves of an AEM electrolyser, recorded for different concentrations of KOH electrolyte, at 60 °C.

The performance and resistance of an AEM electrolyser were investigated further. Specifically, the effect of the liquid electrolyte concentration was determined. Concentrations of 0, 0.1, 0.5 and 1 M KOH were used. The electrolyte flow rate to the anode was maintained at 60 mL min^–1^. The temperature was maintained at 60 °C. Polarization curves of an AEM electrolyser for the various KOH concentrations were recorded. Results are shown in Fig. 7.

The performance curves revealed that the obtained cell voltages decreased with an increase in concentration of the liquid electrolyte. There was a considerable decrease in AEM electrolysis performance when the concentration was reduced from 0.5 to 0.1 M KOH. The obtained cell voltage of electrolysis at 500 mA cm^–2^ was 1.98 V for 1 M KOH, while the cell potential observed for 0.5 M KOH was 2.08 V (higher by 100 mV). These results indicate that the higher concentration of alkaline solution affords very good ionic conductivity of the PBI membrane (in the MEA architecture).

**Figure 8:**
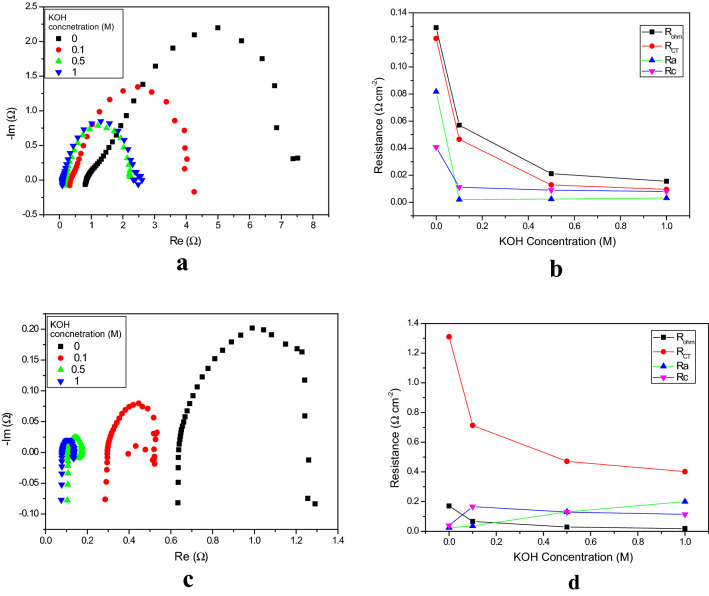
** (a)** Figure 7a Nyquist plots of EIS measurements (from 100 kHz to 1 MHz) for the function of liquid electrolyte concentration at 1.5 V. (**b)** Variation in the relevant electrochemical resistances as a function of concentration at 1.5 V. (**c)** Nyquist plots of the EIS measurements (from 100 kHz to 1 MHz) from the function of liquid electrolyte concertation at 1.9 V. (**d)** Variation in the relevant electrochemical resistances as a function of electrolyte concentration at 1.9 V.

Nyquist plots for liquid electrolyte concentrations, at 1.5 V and 1.9 V, are shown in Fig. 8a,c. The electrochemical resistances recorded for various concentrations are shown in Fig. 8b,d. At 1.5 V, the ohmic resistance also decreased; values of 170.4, 66.8, 28.4 and 18 mΩ cm^–2^ were recorded. When the concentration of KOH was increased from 0 to 1 M at 1.5, the ohmic resistance decreased from 170.4 to 18 mΩ cm^–2^ V. As expected^43^, lower ohmic resistances were recorded under the higher alkaline conditions. One of the main reasons for the lower resistances was increased membrane conductivity, due to the charge transfer, meaning that the ion concentration within the membrane was increased. Osmotic deswelling effects, due to an increase in the concentration of KOH, also contributed additional benefits^12^.

The charge transfer resistance is the major contributing factor to the overall resistance. The charge transfer resistances recorded here were 605, 233, 64 and 48 mΩ cm^–2^ for 0, 0.1, 0.5 and 1 M KOH, respectively. The charge transfer resistance decreased sharply when the concentration of KOH was increased from 0.1 to 0.5 M (77% reduction). However, when the concentration of the electrolyte was increased from 0.5 to 1 M KOH, it was only 25%. At lower cell voltage (1.5 V), the charge transfer resistances are higher than the ohmic resistances. Hence, it appears that at lower KOH concentration the charge transfer resistance dominates, while at higher concentration the ohmic resistance dominates.

With an increase in KOH concentration, the semicircular arc of the Nyquist plot was shifted towards the higher frequency side. The curvature of the Nyquist plot suggests that the catalytic activity of both the OER and HER catalysts has a great effect at the lower concentrations. The ion transport within the catalyst layer and AEM may be associated with an increase in ohmic resistance and charge transfer resistance at lower KOH concentration^44^.

### Effect of temperature on performance and resistances

**Figure 9:**
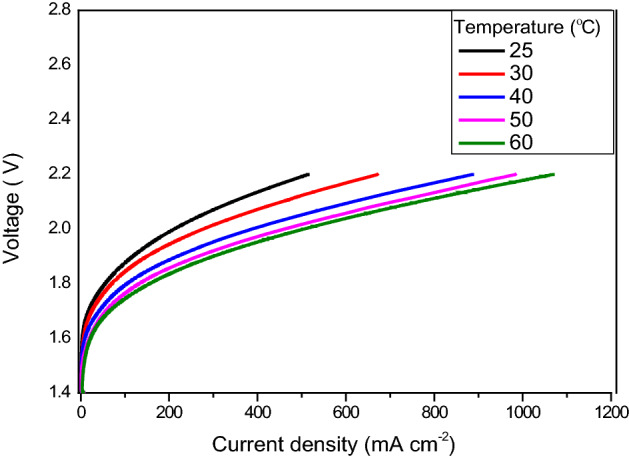
Polarization curves of AEM electrolyser recorded for 1 M KOH at different temperatures.

The effect of different temperatures on the performance of the AEM electrolyser, with 1 M KOH electrolyte, was then investigated. Temperatures of 40, 50 and 60 °C were used and the resulting polarization curves are shown in Fig. 9.

As expected, higher performance was observed at higher temperatures. At 500 mA cm^–2^, the observed cell voltages were 1.98 V and 2.01 V at 60 and 50 °C, respectively. When the electrolyte temperature was reduced to 40 °C, the performance decreased even further: at 500 A cm^–2^ it was 2.1 V. The higher performance at higher temperatures is ascribed to a reduction in ohmic potential and an improvement in catalytic kinetics. At higher temperature, there is a great reduction in the ohmic resistance; at higher temperature the movement of the OH^–^ ions within the membrane is increased, leading to an increase in the rate of reaction. The latter is determined from the diffusion coefficient of OH^–^ ions within the electrode. Furthermore, when the temperature is increased, the catalytic activity also increases, due to rapid electron transfer. Hence, a higher AEM electrolyser performance is achieved at higher temperature.

**Figure 10:**
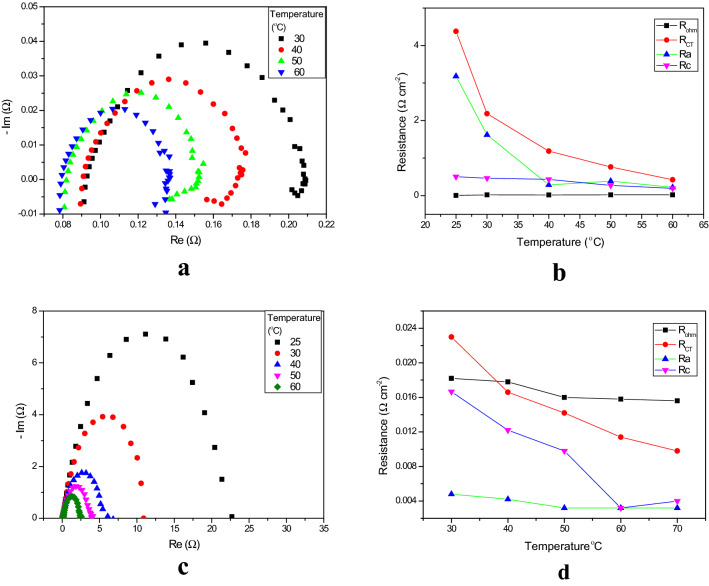
**(a)** Nyquist plots of the EIS measurements (from 100 kHz to 1 MHz) for the function of liquid electrolyte temperature at 1.5 V. (**b)** Variation in the electrochemical resistances as a function of temperature at 1.5 V. (**c)** Nyquist plots of the EIS measurements (from 100 kHz to 1 MHz) for the function of liquid electrolyte temperature at 1.9 V. (**d)** Variation in the electrochemical resistances as a function of temperature at 1.9 V.

Nyquist plots recorded for the various temperature, at 1.5 and 1.9 V, are shown in Fig. 10a,c. The electrochemical resistance recorded for various concentrations, at 1.5 and 1.9 V, is shown in Fig. 10b,d. The ohmic resistance at the higher temperature is lower than at the lower temperature. The obtained ohmic resistances for 30, 40, 50 and 60 °C at 1.9 V are 17.8, 16, 15.8 15.6 mΩ cm^–2^, respectively. For each 10 °C increase in temperature, the increase in ohmic resistance was only 1.2%. The charge transfer resistance for the experimental conditions 40, 50 and 60 °C, at 1.9 V, are 23, 16.6, 14.2 and 11.4 mΩ cm^–2^, respectively The increase in the charge transfer resistance for each 10 °C increase was 8.7%.

The main reason for the reduction in charge transfer resistance is the mobility of the ions within the membrane. At higher temperature, the mobility of the OH^–^ ions is high due to the loosely packed state, the OH^–^ ions can then move faster between the membrane and the catalyst layer. Hence there is an increase in the availability of OH^–^ ions for the redox reaction^43^.

On the other hand, at the lower temperature, the movement of the OH^–^ is slow due to the lower diffusion coefficient. Hence, there is lower availability of ions for the redox reaction, the reason for higher charge transfer resistance. At the low temperature of 30 °C, the contact resistance dominates, whereas when the temperature is increased, the ohmic resistance increases and, subsequently, the charge transfer resistance is reduced. Increasing the temperature to > 60 °C may adversely affect the membrane due to the glass transition temperature of the AEM. To avoid membrane degradation, therefore, an optimum temperature of ~ 60 °C should be maintained for the AEM electrolysis operation.

### Stability of the MEA

Performance and stability are the pillars of any successful water electrolysis technology. Long-term stability is a fundamental requirement of any electrolyser. There are several MEA combinations that exhibit high performance but they have failed, due to a lack of stability^12,44^.

**Figure 11:**
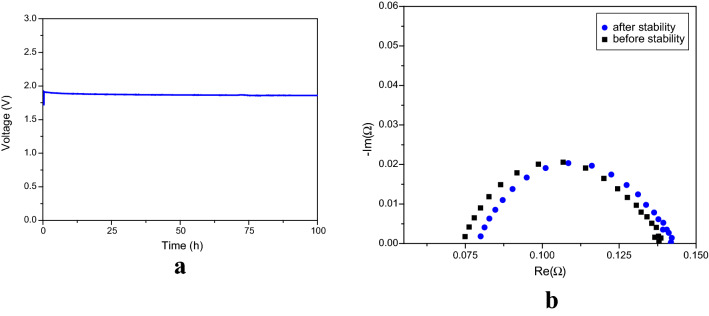
**(a)** Stability evaluation of the MEA over 100 h at 500 mA cm^–2^, at 60 °C. (**b)** Stability determination from the Nyquist plots of the EIS measurements (from 100 kHz to 1 MHz) before and after 100 h.

We determined MEA stability by operating an electrolyser at 500 mA cm^−2^ for 100 h, in constant current mode, in 1 M KOH at 60 °C. Stability analysis was determined from chronoamperomertic analysis and impedance analysis from Nyquist plots. Figure 11a shows the results of the stability evaluation.

Membrane deterioration may be due to the escalation of ohmic resistance and charge transfer resistance. This can be examined using EIS experiments and Nyquist plots. Figure 11b shows a Nyquist plot recorded during an investigation into stability (plots recorded before and after the examination of stability). Ohmic resistance increased with time. The ohmic resistances for the ‘before’ and ‘after’ stability evaluations were 15.9 and 14.8 mΩ cm^–2^, respectively. The increase in ohmic resistance, for both before and after the stability evaluation, was found to be about 1.1 mΩ cm^–2^. The increase in charge transfer resistance was also about 1.1 mΩ cm^–2^. No serious degradation of the catalyst layer nor the membrane was evident.

## Conclusions

The goal of our study was to investigate the various resistances involved in AEM electrolysis, both qualitative and quantitatively, using EIS. More specifically, we wished to determine the contributions of the individual resistances towards the overall resistance and the rate-determining step of AEM electrolysis. We successfully diagnosed and quantified the ohmic and charge transfer resistances from the overall resistances. Furthermore, we demonstrated, for the first time, how the liquid electrolyte flow rate also affects the performance.

At higher flow rates, the removal of OH^–^ ions is very rapid. This reduced the available reaction time for oxidation and reduction, subsequently leading to lower availability of OH^–^ ions for the catalysts, resulting in a significant increase in the overpotentials (especially for the OER). At higher temperature, activation of the catalyst layer expedites the OER reaction, which reduces both the ohmic and charge transfer resistances. Furthermore, an increase in electrolyte concentration increases the availability of OH^–^ ions and the mobility of ions, directly reducing the charge transfer resistance. On the other hand, at lower flow rate, the catalyst active sites are may be blocked by oxygen bubbles.

In all the cases considered here, the cathode resistance was not significantly improved hence, the rate-determining step is the anodic OER process. The optimum working conditions for AEM electrolysis, offering high performance with this MEA, were the following: 1 M KOH liquid electrolyte, temperature 60 °C and flow rate 40 mL min^–1^. Under these conditions, the following was achieved: best performance of 500 mA cm^−2^, with cell potential 1.85 V, and cell resistance as low as 20 mΩ cm^–2^.

Following on from our results to date, suggestions for advancing the design of AEM electrolysers include the following: maintain the optimum flowrate and reduction of anodic ohmic resistance by using plasma-sprayed electrodes. Further research and development in the field of AEM electrolysis is underway in our laboratory, with particular focus on further improving the performance of AEM electrolysis.

## Methods

### Membrane electrode assembly and electrolysis cell setup

The procedures for fabrication of MEA and electrolysis setup were taken from our previous work^17^. The electrolysis reaction was carried out using a specially designed 5 cm^–2^ AEM electrolyser (see Fig. 1b). In the MEA architecture, a PBI membrane was used as the AEM. The MEA was prepared using the catalyst coated on substrate method. The OER and HER catalysts were coated on the Ni foam GDL by spray coating. The AEM was soaked overnight in 1 M KOH, prior to electrolysis, to convert the functional group from Br^–^ into OH^–^. For the anode and cathode, Ni–Fe-O_x_ and Ni–Fe-Co, respectively, coated on the Ni foam diffusion layer, were used. The catalyst loading was 5 mg_cat_ cm^−2^. The MEA was housed between Ti and graphite flow fields. A pair of Teflon gaskets was utilized as a seal, to prevent gas and liquid leakage. Stainless steel bars were used as endplates. The anode and cathode electrical supply probes were connected outside of the endplate. A 500-mL glass tank with circulating water was used as a reservoir. A double-headed peristaltic pump (model: BT 300 FJ CR pump, China) circulated the electrolyte from the reservoir. Preheated 1 M KOH solution was continuously circulated (using a peristaltic pump) through the anode side at a flow rate of 60 mL·min^−1^. The electricity for the electrolysis experiments was supplied through a Potentiostat VSP Biologic instrument (Bio-Logic Science Instruments, Seyssinet-Pariset, France). The temperature was maintained at 60 °C throughout, with a water heating circulating system (F12-ED; Julabo, Germany). The electrolyser was operated under atmospheric pressure. The entire operation was controlled by EC Lab software. The electrochemical impedances were recorded automatically, by computer. The images were created by using Origin(Pro), Version Number (8.5). OriginLab Corporation, Northampton, MA, USA.

### Electrochemical impedance spectroscopy

EIS is an effective analytical technique for an electrochemical system and EIS evaluation is a fairly simple operation. A potentiostat is used to supply a series of low-voltage AC frequencies to a sample and the resulting impedance is recorded. Here, electrochemical impedance spectra were collected using a Potentiostat VSP Biologic instrument and a 10 A booster (Bio-Logic Science Instruments) under potenstiostatic conditions. The AEM electrolyser was directly connected to a potentiostat in two-electrode mode. One electrode acts as the working electrode and the other serves as both reference and counter electrode. The connection wires of the working electrode of the VSP Biologic instrument were connected to the anode current collector. The reference and counter electrodes lead wires were connected to the cathode current collector (see Fig. 1b). Thus, the voltage (sense vs. reference) was measured from the electrolyser terminals/current collectors when current was driven through the working and counter electrodes of the VSP Biologic instrument. EIS investigations were carried out at 1.5 V and 1.9 V, under various conditions, while the frequency was varied from 100 kHz to 100 MHz.

### AEM electrolysis

AEM electrolysis was carried out at 60 °C and a practical application voltage range. The maximum cell voltage was set to 2.2 V. The AC impedance spectra were specifically collected at 1.5 and 1.9 V for lower and higher voltages. The EIS data were collected at 1.5 V identify and quantify the catalytic processes. The data collected at 1.9 V explore the mixed potential such as ohmic, charge transfer and concentration potential affect the performance of the AEM electrolysis system under higher current density.
